# OA-MEN: a fusion deep learning approach for enhanced accuracy in knee osteoarthritis detection and classification using X-Ray imaging

**DOI:** 10.3389/fbioe.2024.1437188

**Published:** 2025-01-03

**Authors:** Xiaolu Ren, Lingxuan Hou, Shan Liu, Peng Wu, Siming Liang, Haitian Fu, Chengquan Li, Ting Li, Yongjing Cheng

**Affiliations:** ^1^ Department of Radiology, General Hospital of Ningxia Medical University, Yinchuan, China; ^2^ School of Health Sciences, Universiti Sains Malaysia, Kota Bharu, Kelantan, Malaysia; ^3^ College of Biomedical Engineering, Sichuan University, Chengdu, Sichuan, China; ^4^ Department of Orthopedics, General Hospital of Ningxia Medical University, Yinchuan, China; ^5^ School of Clinical Medicine, Tsinghua University, Beijing, China; ^6^ Department of Rheumatology and Immunology, Beijing Hospital, National Centre of Gerontology, Beijing, China

**Keywords:** knee osteoarthritis, deep learning, decision making, artificial intelligence, convolution natural network

## Abstract

**Background:**

Knee osteoarthritis (KOA) constitutes the prevailing manifestation of arthritis. Radiographs function as a common modality for primary screening; however, traditional X-ray evaluation of osteoarthritis confronts challenges such as reduced sensitivity, subjective interpretation, and heightened misdiagnosis rates. The objective of this investigation is to enhance the validation and optimization of accuracy and efficiency in KOA assessment by utilizing fusion deep learning techniques.

**Methods:**

This study aims to develop a highly accurate and lightweight model for automatically predicting and classifying KOA through knee X-ray imaging. We propose a deep learning model named OA-MEN, which integrates a hybrid model combining ResNet and MobileNet feature extraction with multi-scale feature fusion. This approach ensures enhanced extraction of semantic information without losing the advantages of large feature maps provided by high image resolution in lower layers of the network. This effectively expands the model’s receptive field and strengthens its understanding capability. Additionally, we conducted unseen-data tests and compared our model with widely used baseline models to highlight its superiority over conventional approaches.

**Results:**

The OA-MEN model demonstrated exceptional performance in tests. In the unseen-data test, our model achieved an average accuracy (ACC) of 84.88% and an Area Under the Curve (AUC) of 89.11%, marking improvements over the best-performing baseline models. These results showcase its improved capability in predicting KOA from X-ray images, making it a promising tool for assisting radiologists in diagnosis and treatment selection in clinical settings.

**Conclusion:**

Leveraging deep learning for osteoarthritis classification guarantees heightened efficiency and accuracy. The future goal is to seamlessly integrate deep learning and advanced computational techniques with the expertise of medical professionals.

## 1 Introduction

The rising global prevalence of osteoarthritis (OA), especially in the knee joint, poses a significant public health challenge (Latourte et al., 2020). OA is typically triggered by a combination of subtle risk factors present in daily life, including diet, hormonal levels, and genetic predispositions. The incidence of OA significantly increases with age (Jia et al., 2023). A recent Chinese meta-analysis involving 74,908 symptomatic patients revealed an overall Knee osteoarthritis (KOA) prevalence of 14.6% between 2012 and 2016, with higher rates among females (19.1%) compared to males (10.9%) (Li et al., 2020). KOA varies in its progression, with the majority of patients seeing gradual deterioration over decades, while some experience rapid decline. Moreover, the onset of KOA often coincides with other comorbidities, exacerbating its impact and ultimately leading to the necessity for knee replacement surgery in some cases (Kotela et al., 2019; Wojdasiewicz et al., 2020; Wu et al., 2022). Early and accurate diagnosis is vital for effective management and timely intervention in KOA cases (Wojdasiewicz et al., 2020).

X-ray imaging is indispensable for the diagnosis of KOA, providing a non-invasive and cost-effective means to assess joint damage and monitor disease progression (Hirvasniemi et al., 2019; Jansen et al., 2021). In clinical practice, physicians rely on the Kellgren-Lawrence (K-L) grading system (Kellgren and Lawrence, 1957), designed for visual inspection of X-ray images, to measure the severity of KOA. The K-L system categorizes KOA severity into five grades, ranging from grade 0 (normal) to grade 4 (severe). While K-L grading methods are valuable tools for clinicians, the clinical assessment of conditions like KOA faces inherent challenges marked by subjectivity, the lack of quantitative data, and the absence of standardization (Abdullah and Rajasekaran, 2022; Cueva et al., 2022). This subjectivity leads to varying interpretations among healthcare practitioners, resulting in inconsistent diagnoses and treatment recommendations. The absence of quantitative data, combined with the lack of standardization in assessment techniques and protocols across healthcare providers, exacerbates these disparities, making it difficult to effectively monitor changes over time or evaluate the effectiveness of different treatment approaches. Consequently, the development of a fully automatic and efficient auxiliary classification method becomes imperative.

The rise of artificial intelligence technology has prompted extensive research into the application of deep learning in arthritis diagnosis. Deep learning, particularly convolutional neural networks (CNNs), excels at automatic feature extraction from intricate data, enabling the identification of subtle differences that may challenge human perception. This capability positions deep learning as a potent tool for tasks like image recognition and classification (Panwar et al., 2020). Deep learning has proven effective in various knee-related applications, including the diagnosis of bone tumors (Do et al., 2021), identification of knee joint injuries (Kim et al., 2022; Qu et al., 2022; Wang et al., 2023), and the recognition and segmentation of anatomical structures (Gan et al., 2021; Quinsten et al., 2022). Concurrently, numerous studies concentrate on employing deep learning for knee grading, showcasing diverse techniques for automated KOA severity grading from X-ray images. Chen et al. (2019) utilized YOLO2 and fine-tuned VGG-19, achieving 69.7% accuracy with a Mean Absolute Error of 0.344. Thomas et al. (2020) developed a DenseNet with an accuracy of 0.71 and an F1 score of 0.70 for KOA grading. Wang et al. (2021) integrated YOLO with a visual transformer, achieving 69.18% accuracy in diagnosis. Cueva et al. (2022) present a CADx model employing Deep Siamese CNNs and fine-tuned ResNet-34 for simultaneous detection of OA lesions in both knees (KL scale), achieving 61% average multi-class accuracy. Mohammed et al. (2023) attained peak classification accuracies of 69%, 83%, and 89%, respectively, utilizing the ResNet101 deep neural network (DNN) model. However, the diagnosis of KOA primarily relies on texture features, and general models exhibit limited capability in recognizing these features, resulting in lower accuracy rates that fail to meet clinical application standards. Hence, there is a pressing need to develop a highly accurate model for the automatic diagnosis and grading of KOA. Such a model would assist radiologists in making more accurate diagnoses, reduce the rate of misdiagnosis, and consequently minimize patient suffering.

In this study, we developed a deep learning model named OA-MEN, which is a fusion model obtained by integrating multi-scale feature fusion with parallel ResNet and MobileNet architectures. The multi-scale feature fusion within the ResNet structure effectively increases the network depth, combining the high-level semantic expression capabilities of the upper network layers with the geometric information representation of the lower layers. This integration expands the model’s receptive field and utilizes residual blocks to prevent gradient vanishing, thereby avoiding ineffective training. MobileNet enhances the model’s comprehension ability by extracting more effective features without significantly increasing model complexity. The final prediction and output are conducted through a fully connected layer. Tests with unseen data and comparisons with baseline models demonstrate this method’s effectiveness in enhancing the model’s performance and understanding ability for multi-classification of osteoarthritis.

## 2 Materials and methods

**FIGURE 1:**
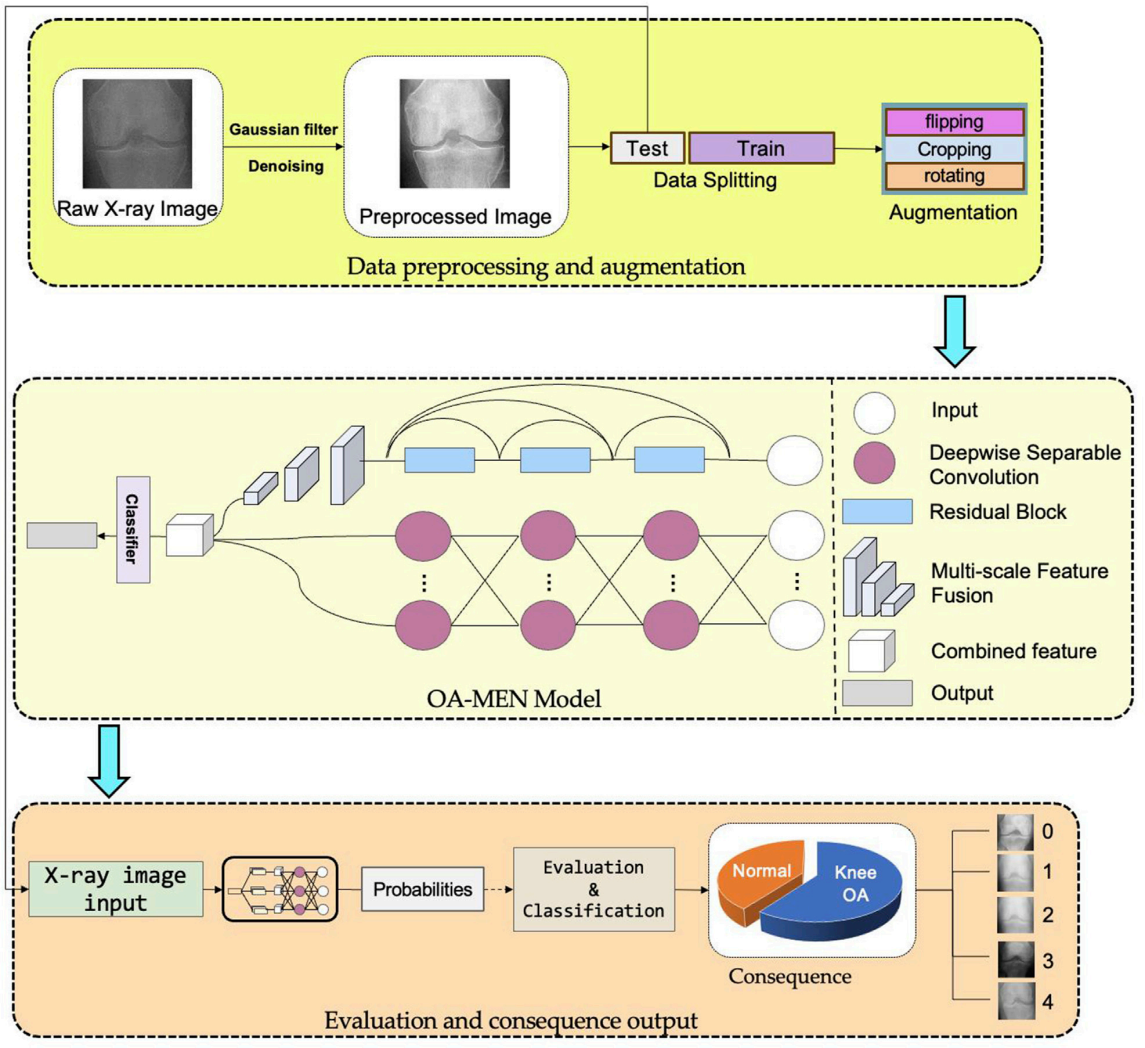
The workflow of this study. All original images undergo preprocessing steps such as denoising and detail enhancement before being divided into training and testing datasets. The training set, after data augmentation, is fed into the OA-MEN model for model training. The trained model is then evaluated using the test set.

The workflow of this study is shown in Figure 1.

### 2.1 Database

**FIGURE 2:**
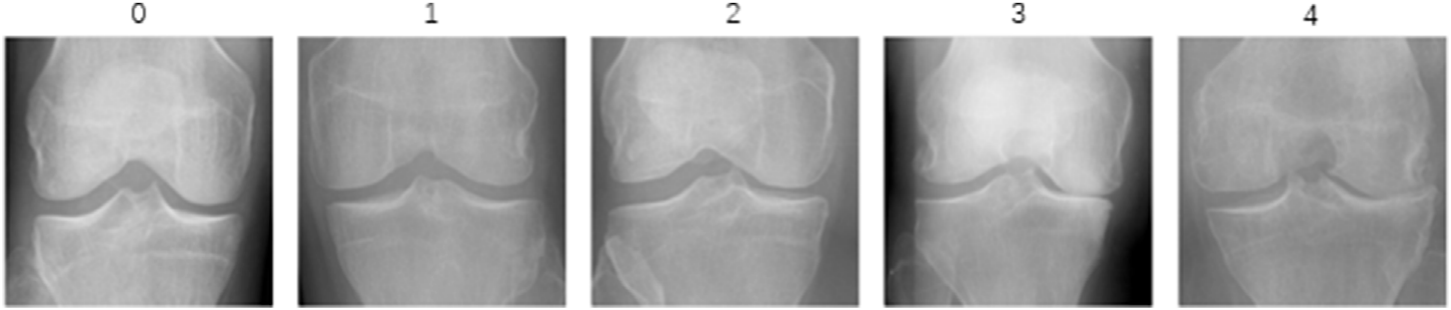
The diagram of KOA Severity Grading Dataset. The image demonstrates increasing severity from left to right, starting with a normal joint in Grade 0 and advancing through slight changes in Grade 1, evident osteophytes in Grade 2, significant joint space narrowing and sclerosis in Grade 3, to severe joint space loss and bone deformity in Grade 4.

In this study, we utilized the KOA Severity Grading Dataset from Kaggle (Chen, 2018) as our training and testing data source. This dataset comprises 9786 high-quality knee X-ray images, each labeled by clinical experts according to the knee Kellgren-Lawrence (KL) grading standards (Kellgren and Lawrence, 1957). The KL grading system is a widely used radiological classification system for assessing the severity of KOA. Initially proposed by Kellgren and Lawrence in 1957, it primarily focuses on X-ray features to evaluate the progression and severity of KOA. The KL grading system categorizes KOA into levels as outlined in Table 1. The diagram of KOA Severity Grading Dataset is shown in Figure 2, which illustrates the progression of knee osteoarthritis through the KL grading system. Grade 0 shows no signs of osteoarthritis with a normal joint appearance. Grade 1 reveals slight narrowing of the joint space and possible osteophytic lipping. Grade 2 displays definite osteophytes and possible narrowing of the joint space. Grade 3 shows moderate multiple osteophytes, definite narrowing of the joint space, some sclerosis, and possible deformity of bone contour. Grade 4 exhibits large osteophytes, marked narrowing of the joint space, severe sclerosis, and definite deformity of bone contour.

**TABLE 1:** The knee K-L grading system.

Grade	Description
0	Healthy knee image
1 (Doubtful)	Doubtful joint narrowing with possible osteophytic lipping
2 (Minimal)	Definite presence of osteophytes and possible joint space narrowing
3 (Moderate)	Multiple osteophytes, definite joint space narrowing, with mild sclerosis
4 (Severe)	Large osteophytes, significant joint narrowing, and severe sclerosis

### 2.2 Data preprocessing

In the initial stages of the experiment, we conducted preprocessing operations on all training images. Firstly, we applied a Gaussian filter to the training set images to remove high-frequency noise, followed by edge detection using the Laplacian operator for edge feature extraction, which was then combined with the original images to enhance the representation of texture features. Additionally, histogram equalization was performed in the brightness space to augment the display of image brightness and details. Moreover, the original dataset was divided into training and testing sets in an 8:2 ratio for conducting unseen-data tests.

Given the presence of data imbalance in the original dataset and to enhance the model’s ability to recognize anomalous inputs, we employed data augmentation techniques for the training set. For each category, we increased the number of images through cropping, flipping, and translation, equalizing the image quantity across categories to prevent model overfitting due to data imbalance. For fair comparison, all baseline models used datasets that had undergone similar data augmentation and preprocessing. Tests showed that such preprocessing successfully improved the model’s predictive performance.

### 2.3 Model construction

#### 2.3.1 OA-MEN model

In our preliminary experiments, it was observed that most deep learning models lacked the capability to effectively extract texture information in KOA recognition, thereby limiting their performance. To address this issue, we propose the OA-MEN model, a fusion of multi-scale feature-integrated ResNet and MobileNet. ResNet, introduced by He et al. (2016) in 2015, achieves this goal through the incorporation of “residual blocks.” Within these blocks, the input is not only passed to the next layer but also directly through skip connections to deeper layers. This architecture allows the network to learn residuals between inputs and outputs, rather than learning the outputs directly, facilitating the construction of deeper models without encountering the problem of gradient vanishing. MobileNet, developed by Howard et al. (2017) from Google in 2017, is centered around depthwise separable convolutions, which decompose traditional convolution into depthwise and pointwise convolutions. Depthwise convolution applies individual filters to each input channel, while pointwise convolution, a 1 × 1 convolution, combines the output of depthwise convolutions. This approach significantly reduces the model’s complexity and computational demands while maintaining robust performance. As another branch of our model, MobileNet enhances the extraction of detailed features without substantially increasing computational load. To enhance the recognition of critical texture features in KOA, the model utilizes multi-scale feature fusion, capturing fine surface details (acquired in shallower network layers) and more abstract texture patterns (captured in deeper layers).

**FIGURE 3:**
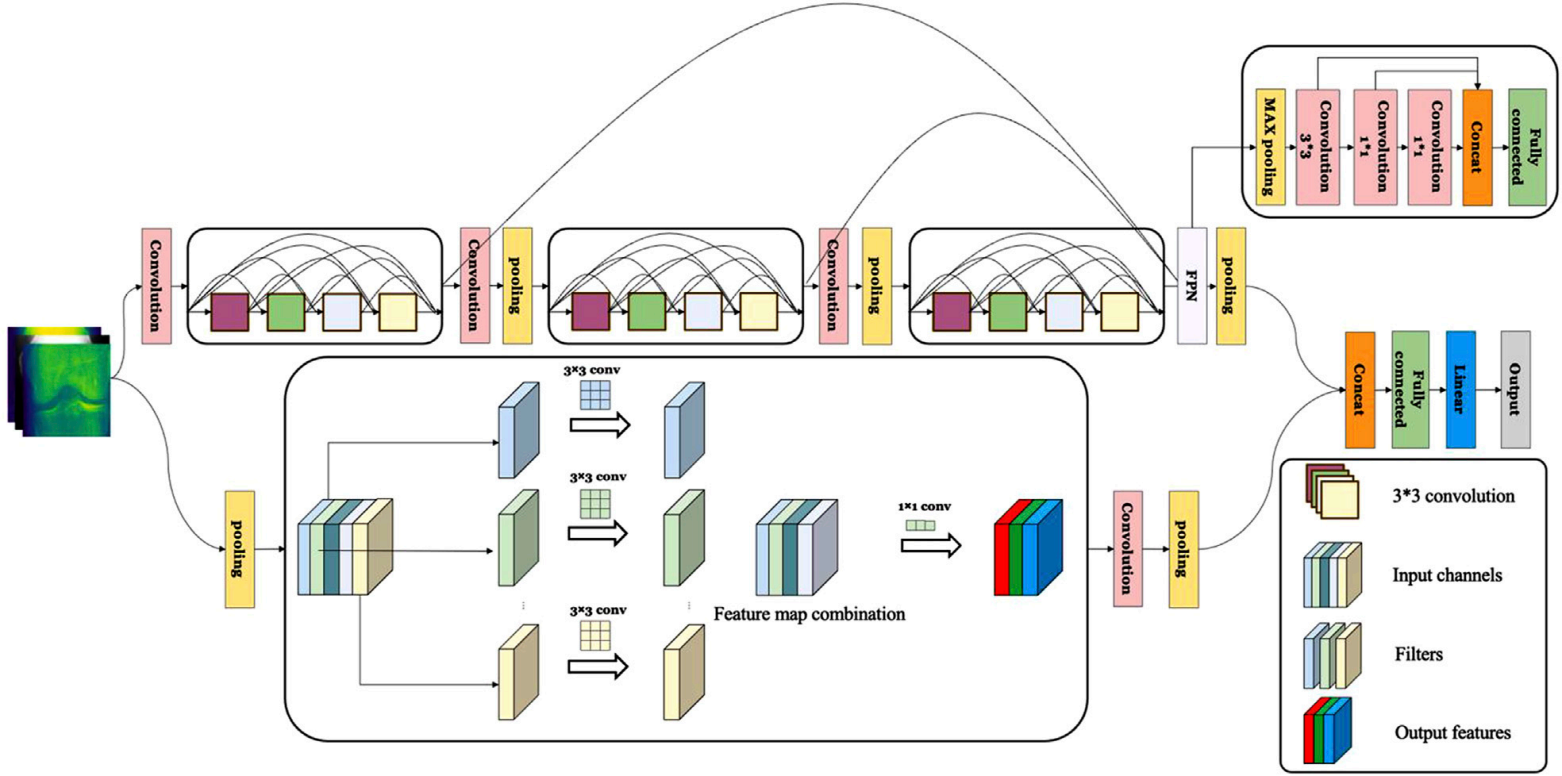
The construction of OA-MEN model. The model integrates a ResNet-based structure with multi-scale feature fusion, enhancing the network depth and combining rich semantic information with larger feature maps. In parallel, MobileNet is employed to further extract and complete feature representations, significantly enhancing the model’s interpretative capability.

In summary, the ResNet module in the OA-MEN model allows for the construction of deeper learning architectures and avoids gradient vanishing, laying the groundwork for multi-scale feature fusion to extract information at various levels. The multi-scale feature fusion module effectively extracts both fine surface details and elusive texture features across different layers and scales. Furthermore, MobileNet, as a complementary branch, captures detailed features without significantly increasing computational load. These modules work in synergy to not only extract surface details from Knee Joint X-ray images but also effectively capture texture features, enhancing the model’s ability to diagnose and classify KOA. The model construction of OA-MEN is shown in Figure 3.

#### 2.3.2 Comparison models

To objectively evaluate the performance of the OA-MEN model proposed in this study, we employed traditional deep learning models (Xception, MobileNet, ResNet, DenseNet, and NasNet) for comparison. These models have been widely applied by researchers and have demonstrated their superior performance and reliability, serving as benchmarks and future directions for our research. The Xception model, an evolution of the Inception (Szegedy et al., 2015) architecture, was introduced by Chollet in 2016. It utilizes depthwise separable convolutions within the Inception module, enabling the network to learn spatial hierarchies of features more efficiently while reducing the model’s parameters and computational complexity. Xception has shown excellent performance in various image recognition tasks and has been increasingly applied in KOA recognition in recent years (Sindhu et al., 2022; Abd El-Ghany et al., 2023).

DenseNet, proposed by Huang et al., in 2016, features a densely connected pattern where each layer is directly connected to all preceding layers, effectively improving information and gradient flow and enhancing parameter efficiency. DenseNet has been extensively proven effective in various image classification and recognition tasks, including medical imaging and KOA recognition (Chaugule and Malemath, 2022; Alexopoulos et al., 2023).

NASNet, a neural network architecture designed using automated machine learning techniques, was introduced by Google Brain researchers Zoph et al. (2018) in 2018. NASNet is a product of Neural Architecture Search (NAS), wherein machine learning autonomously discovers high-performance network architectures. Exhibiting outstanding performance in multiple standard image recognition benchmarks, NASNet represents a trend in discovering neural network architectures through automated methods and has also been applied in medical imaging and KOA recognition (Sultan et al., 2021; Yoon et al., 2023).

### 2.4 Model evaluation

To evaluate our OA-MEN model, we used several key performance metrics: Accuracy (ACC), Precision (PRE), Recall (REC), and Area Under the ROC Curve (AUC). These metrics are derived from the counts of True Positives (TP), True Negatives (TN), False Positives (FP), and False Negatives (FN), which reflect correct or incorrect predictions by the model. The formulas for calculating ACC, PRE, REC, and F1 are as follows (Equations 1–4):

$$ACC=\frac{TP+TN}{TP+FP+TN+FN}$$ (1)

$$PRE=\frac{TP}{TP+FP}$$ (2)

$$REC=\frac{TP}{TP+FN}$$ (3)

$$F1=\frac{2\times PRE\times REC}{PRE+REC}$$ (4)

The ROC curve plots the trade-off between the True Positive Rate (TPR) and False Positive Rate (FPR) at various thresholds, with the AUC indicating the model’s ability to distinguish between classes. An AUC close to 1 suggests high effectiveness, while an AUC near 0.5 implies random performance.

## 3 Results

### 3.1 Experimental setup

In this study, we implemented uniform parameter optimization across all models to ensure an objective evaluation. We employed the gradient threshold method for tuning all parameters. After extensive experimentation and testing, we chose the suitable number of epochs to make the models to fully converge without exhibiting signs of overfitting. The learning rate was set correctly to facilitate appropriate convergence speed and mitigated the risk of settling at local optima.

The experiments in this study were conducted on a system operating with Windows 11 Professional, utilizing Python 3.10.9 as the programming environment. In terms of software libraries, we employed Pytorch 2.0.1 + cu117, Scikit-learn, Sklearn 0.0.post1, and scipy 1.10.0, along with other mathematical libraries, to facilitate the development of the model’s architecture and the validation of its results. The hardware setup encompassed an Intel Core i7 10750H processor, featuring a base frequency of 2.6 GHz and a turbo frequency of up to 5 GHz, with 6 cores and 12 threads. Additionally, the system was equipped with an NVIDIA GeForce GTX 1080Ti graphics card, which has an 8 GB memory capacity and a 128-bit memory bus width.

### 3.2 Model results

**FIGURE 4:**
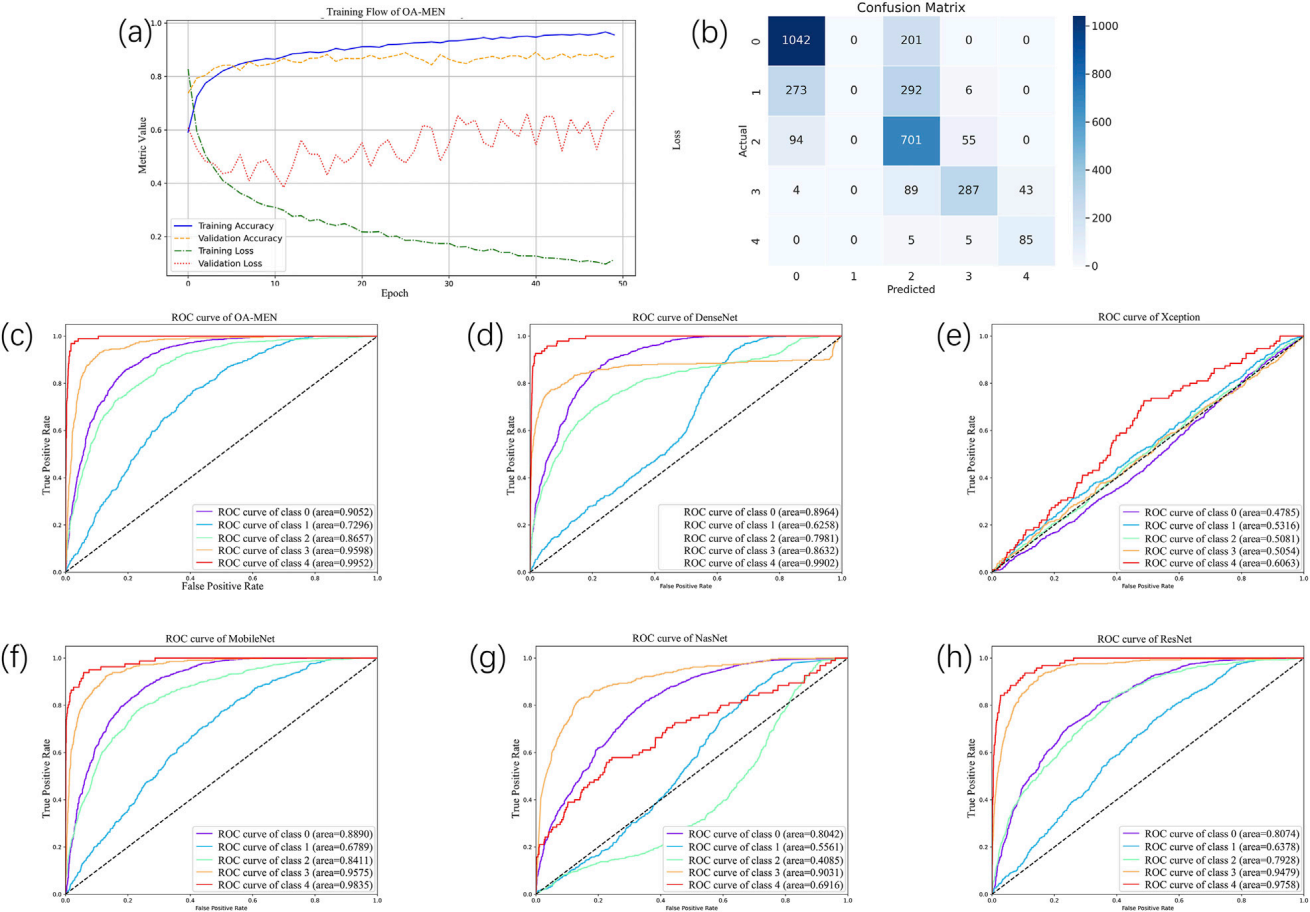
The result of all models. **(A)** The training flow of OA-MEN model. As illustrated by the curve, the model converges and achieves optimal results after 50 training epochs. **(B)** The confusion matrix of OA-MEN model. The model accurately predicts most categories; however, the classification of Kellgren-Lawrence Grade 1 (doubtful osteoarthritis) poses challenges, making it difficult to distinguish between Grades 0 and 2. **(C–H)** The ROC of OA-MEN, DenseNet, Xception, MobileNet, NasNet and ResNet. The ROC curve for the OA-MEN model is the closest to the top-left corner, indicating the highest AUC across all categories compared to the other models, thereby demonstrating superior predictive performance.

To conduct a more comprehensive and objective evaluation of the predictive performance of the model proposed in this paper, we divided the dataset into training and testing sets in an 8:2 ratio. The testing set, serving as unseen data, was utilized to assess the model’s predictive capabilities on datasets it had not previously encountered. Through experimental validation, we ultimately determined that training the model for 50 epochs with a learning rate of 0.00001 allowed for complete convergence without signs of overfitting. The iteration graph of the OA-MEN model is depicted in Figure 4A.

In the unseen-data test, the OA-MEN model exhibited outstanding performance. Specifically, for mild KOA (class 1), the model proposed in this study achieved a classification accuracy of 75.87%, the highest compared to all traditional models. In terms of average ACC and AUC, the OA-MEN model reached 84.88% and 89.11%, respectively, as shown in Table 2. Such high-performance metrics position the OA-MEN model as a promising tool for deployment in clinical settings, assisting radiologists in diagnosis. The confusion matrix and ROC curve of the OA-MEN model are illustrated in Figures 4B, C.

**TABLE 2:** The performance of all models.

	Mean ACC	Mean PRE	Mean REC	Mean AUC
OA-MEN	84.88	63.57	64.03	89.11
MobileNet	83.50	65.07	61.60	87.00
Xception	80.69	51.66	60.52	83.47
ResNet	83.86	60.13	62.71	83.23
DenseNet	83.62	59.09	60.68	87.56
NasNet	64.78	40.09	24.33	67.27

Among the comparison models, ResNet demonstrated the best performance, with an average ACC and AUC of 83.86% and 83.23%, respectively. However, there was a significant gap between ResNet and the OA-MEN model, particularly in the classification accuracy for mild KOA, where ResNet achieved only 73.88%. This discrepancy could potentially mislead radiologists in selecting appropriate treatment methods and impose unnecessary burdens on patients. The ROC curves for the comparison models are depicted in Figures 4D–H.

### 3.3 Model visualization and clinical interpretability

Most grade 0 Grad-CAM heatmaps do not exhibit a distinct gradient color heatmap compared to cases of higher-grade osteoarthritis, suggesting that the model’s focus may not be primarily on specific areas associated with pathological features in this non-osteoarthritis category. Instead, accurate predictions of grade 0 may be attributed to a more extensive and precise assessment of normal anatomy, aligning with clinical expectations of a lack of significant pathological manifestations. The gradient color heatmaps detected in a limited number of level 0 images suggest subtle changes or features capturing the model’s attention. This prompts the intriguing hypothesis that these images may not be genuinely normal and could potentially exhibit some pathological alterations. The distribution of most heatmaps for grades 1 to 4 aligns with the distribution of narrow joint spaces. However, the eye-catching appearance of the gradient color heatmaps of the subarticular area, combined with previous studies, leads to the proposal of a second interesting hypothesis that altered remodeling of subchondral trabecular bone may attract the attention of deep learning models. All of this necessitates future research to incorporate this potential hypothesis into the study design for further investigation and validation.

**FIGURE 5:**
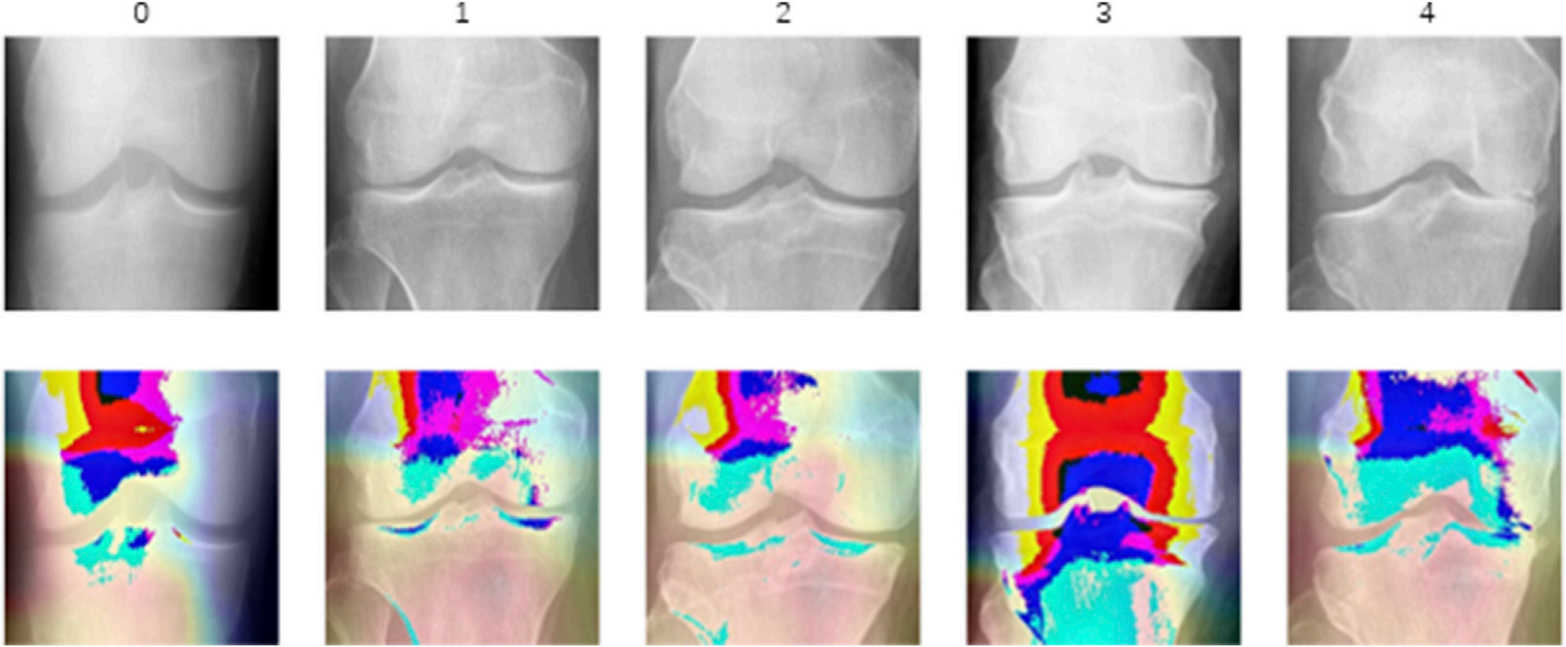
The heat map of different categories. These heat maps use color intensity to indicate regions of significant degenerative change, with warmer colors typically representing higher significance or more severe degradation.

Visualization of heat maps enhances the interpretability of deep learning models but also presents certain challenges, especially compared to traditional imaging biomarkers. The heat map of five categories is shown in Figure 5. Heat maps capture static snapshots of decisions made at a given moment, providing a partial view of the model’s attention and visualizing the features the model learns. Grade 0 displays minimal color variation, indicating an absence of significant osteoarthritic changes, which corroborates with the absence of OA. Grade 1 shows slight color changes around the joint space, suggesting the model’s early detection of potential osteoarthritic alterations. Grade 2 is characterized by increased color intensity near the joint edges, highlighting early degenerative changes such as minor joint space narrowing and possible osteophyte formation. Grade 3 exhibits pronounced warm colors in the joint space and surrounding bone, signifying moderate reductions in joint space, osteophyte growth, and potential sclerosis. Grade 4 focuses on the bone itself, where the model pays particular attention to changes in bone morphology and density as well as the formation of osteophytes. However, their display may not be consistent with conventional imaging biomarkers that clinicians are familiar with. Deep learning models, including those that use heat maps, often have black-box properties, making the internal decision-making process not completely transparent. Additionally, these models may be inherently uncertain, and heat maps may not clearly convey the confidence or uncertainty of the model’s predictions. These limitations may affect the interpretation process and results, necessitating increased adoption of these novel visualization techniques by radiologists and clinicians and further exploration of interpretability.

## 4 Discussion

Aiming to enhance the detection accuracy of KOA and reduce the misdiagnosis and underdiagnosis of mild cases, this paper introduces a novel deep learning model, named the OA-MEN model. It is a fusion model, combining ResNet with multi-scale feature integration and MobileNet in parallel. This model achieves accurate detection of KOA by synthesizing surface information and detailed texture features from feature maps of various network depths, thus increasing the detection accuracy for mild KOA and overall.

The OA-MEN model leverages the ResNet architecture to increase network depth without encountering issues like gradient vanishing. It integrates the semantic expressiveness of higher-level network structures with the geometric representational ability of lower-level structures through multi-scale feature fusion, thereby expanding the model’s receptive field. In parallel, MobileNet extracts more effective features without significantly increasing the model’s complexity, enhancing its understanding capability. The model culminates in a fully connected layer for prediction and output. Therefore, it effectively captures texture features and surface details of KOA, enhancing the model’s performance and understanding of the multi-classification of osteoarthritis.

Despite positive outcomes in existing studies, this investigation in the field of precise and automatic osteoarthritis grading aims to enhance, validate, and extend the applicability of findings. Acknowledging positive outcomes from previous studies, the research maintains a rigorous approach, emphasizing the necessity for validation across diverse datasets and external test data. This work not only advances the current understanding of osteoarthritis grading but also sets the stage for future refinements, multi-center validations, and the development of a deployable clinical tool, demonstrating an ongoing innovation in automated medical grading systems.

Despite the promising implications of our study, our grading system has limitations. Limited external test data from a specific cohort impacts generalizability. Future research should include diverse data from multiple centers. Furthermore, the KL grading system presents difficulties in differentiating between KL grade 1 (doubtful OA) and other grades due to its definition and the limited data available, an issue our model has not yet resolved. Future research should aim to increase the training samples and enhance the model to address this challenge (Zhao et al., 2024). Also, our system is not yet deployable for clinical use, it necessitates additional development. Moreover, our study lacks external validation, which is critical for ensuring the robustness and generalizability of our proposed model. It is essential to conduct further testing on more diverse datasets and to acquire clinical samples of KOA to demonstrate the superiority and clinical applicability of our model. Future research can refine our findings, contributing to a sophisticated and clinically applicable osteoarthritis grading system.

## 5 Conclusion

This study aims to precisely predict and grade KOA, introducing the OA-MEN model, a hybrid deep learning model that leverages ResNet and a multi-scale feature fusion strategy. This approach enhances the model’s ability to extract texture features while capturing rich information from high-resolution feature maps. Additionally, the integration of a parallel MobileNet allows the model to extract more effective features without substantially increasing its complexity, thus augmenting the model’s overall understanding of KOA characteristics. Through unseen-data testing and comparison with traditional models, the OA-MEN model has demonstrated superior performance. Using the Knee KL grading system for classification and grading, the model achieved average ACC and AUC of 84.88% and 89.11%, respectively, fulfilling the objective of accurately predicting KOA. Further validation and optimization of this study will enhance the translation of these promising results into practical applications. Future research should encompass a more diverse array of external test data, employ more sophisticated methodologies, undergo further development and validation, and transition from a research setting to a clinically applicable tool. Looking forward, this study is expected to be deployed in clinical settings to assist physicians in diagnosis, reduce misdiagnosis rates, and minimize the patient harm caused by misdiagnoses and underdiagnoses.

## Data Availability

Publicly available datasets were analyzed in this study. This data can be found here: P. J. M. D. Chen, Knee osteoarthritis severity grading dataset, 1 (2018) 21-23.
