# Supplementary material for: OA-MEN: a fusion deep learning approach for enhanced accuracy in knee osteoarthritis detection and classification using X-Ray imaging
Source: Front Bioeng Biotechnol. 2025 Jan 3;12:1437188. doi: 10.3389/fbioe.2024.1437188 (PMC11739149; doi:10.3389/fbioe.2024.1437188)
Supplement: Supplementary file 1 [file Image2.pdf]

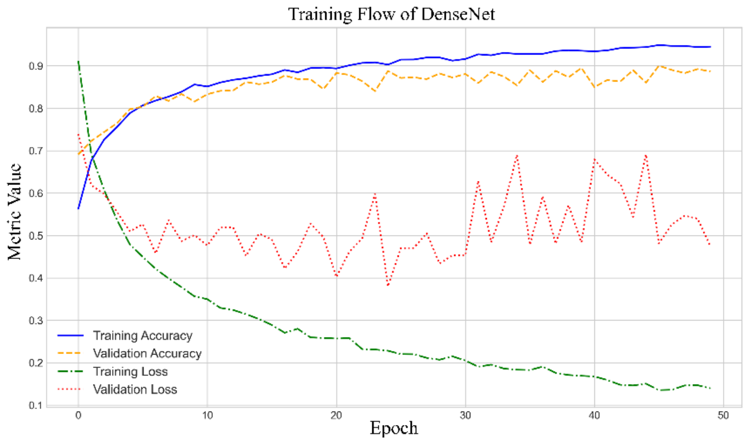


Fig. a. The training flow of DenseNet

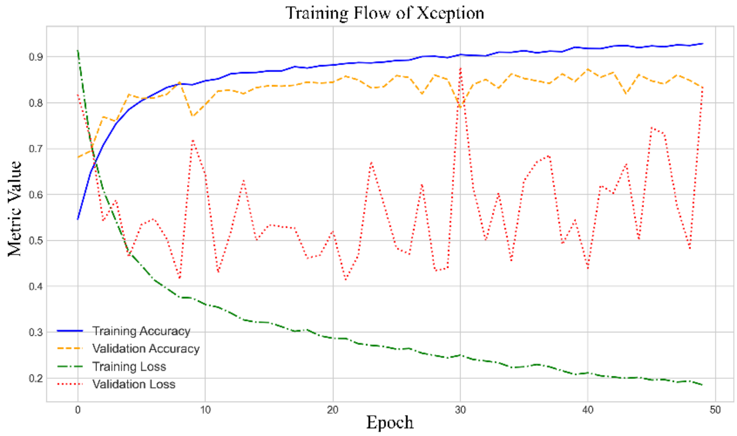


Fig. b. The training flow of Xception

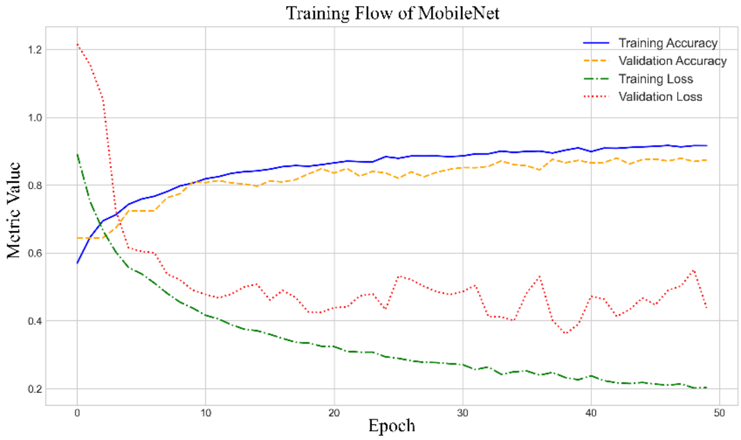


Fig. c. The training flow of MobileNet

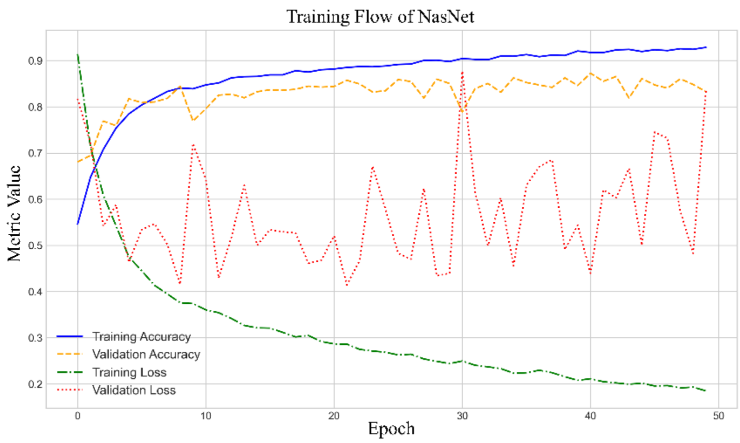


Fig. d. The training flow of NasNet

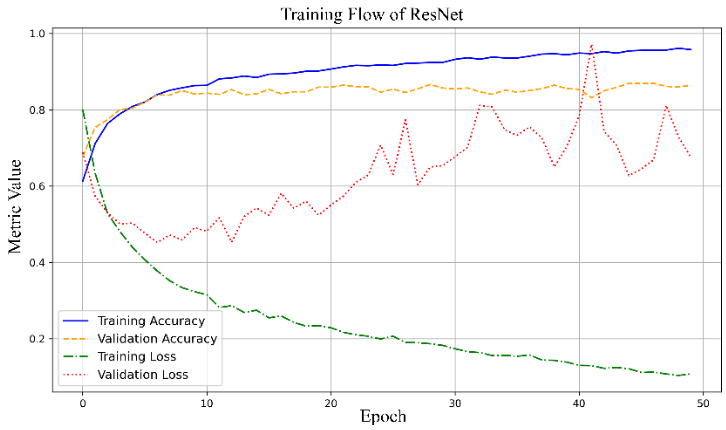


Fig. e. The training flow of ResNet
