# Supplementary material for: OA-MEN: a fusion deep learning approach for enhanced accuracy in knee osteoarthritis detection and classification using X-Ray imaging
Source: Front Bioeng Biotechnol. 2025 Jan 3;12:1437188. doi: 10.3389/fbioe.2024.1437188 (PMC11739149; doi:10.3389/fbioe.2024.1437188)
Supplement: Supplementary file 2 [file Image1.pdf]

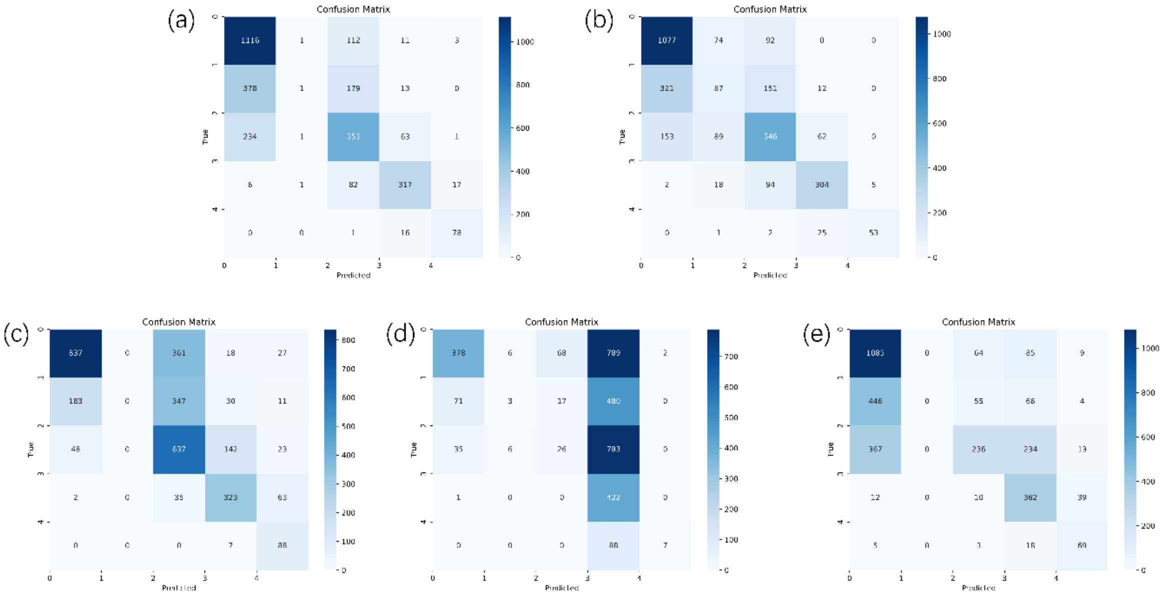

Supplementary Figure 1 (a)-(e) the confusion matrix of Xception, NasNet, ResNet, DenseNet and MobileNet
